# Tick-borne pathogens and associated co-infections in ticks collected from domestic animals in central China

**DOI:** 10.1186/1756-3305-7-237

**Published:** 2014-05-22

**Authors:** Zhuo Chen, Qin Liu, Ji-Qi Liu, Bian-Li Xu, Shan Lv, Shang Xia, Xiao-Nong Zhou

**Affiliations:** 1 National Institute of Parasitic Diseases, Chinese Center for Disease Control and Prevention, Shanghai 200025, People’s Republic of China; 2 Schistosomiasis and Filariasis; Key Laboratory of Parasite and Vector Biology, Ministry of Health, WHO Collaborative Center for Malaria, Shanghai 200025, People’s Republic of China; 3 Henan Center for Disease Control and Prevention, Zhengzhou 450016, People’s Republic of China

**Keywords:** Ticks, Domestic animals, Tick-borne pathogens, Co-infections, China

## Abstract

**Background:**

Ticks can transmit a number of pathogens to humans and domestic animals. Tick borne diseases (TBDs), which may lead to organ failure and death have been recently reported in China. 98.75% of the total cases (>1000) in Henan provinces have been reported in Xinyang city. Therefore, the aims of this study were to investigate the fauna of ticks and detect the potential pathogens in ticks in Xinyang, the region of central China.

**Methods:**

Ticks were collected from 10 villages of Xinyang from April to December 2012, from domestic animals including sheep, cattle and dogs. Then identification of ticks and detection of tick-borne pathogens, including *Babesia* spp.*, Theileria* spp.*, Anaplasma* spp.*, Ehrlichia* spp.*, Rickettsia* spp.*,* tick-borne encephalitis virus (TBEV), *Borrelia burgdorferi sensu lato, Leishmania infantum,* were undertaken by using polymerase chain reaction assay (PCR) and sequence analysis. Moreover, the co-infection patterns of various pathogens were compared among locations where ticks were collected.

**Results:**

A total of 308 ticks were collected. Two species of Ixodidae were found, namely *Haemaphysalis longicornis* (96.75%) and *Rhipicephalus microplus* (3.25%)*.* Five genera of pathogens, namely *Theileria* spp. (3.25%), *Anaplasma* spp. (2.92%), *Babesia* spp. (1.95%), *Ehrlichia* spp. (2.92%) and *Rickettsia* spp. (0.65%), were detected in 7 villages. Co-infections by two pathogens were diagnosed in 11.11% of all infected ticks.

**Conclusions:**

Both human and animal pathogens were abundant in ticks in the study areas. Humans and animals in these regions were at a high risk of exposure to piroplasmosis, since piroplasm had the highest rates of infection and co-infection in positive ticks.

## Background

Ticks (Acari: Ixodida) are parasitic acari that suck blood from their vertebrate hosts [1]. They can transmit a number of pathogenic organisms to humans and domestic animals [2,3] and cause a variety of important natural focal diseases and zoonoses. Ticks are important pests and vectors of several pathogens in tropical and subtropical regions [4-6].

To date, more than 900 species of ticks have been recorded globally, with two major families, namely Ixodidae and Argasidae, the former generally referred to as hard ticks and the later also known as soft ticks [7]. In China, about eleven genera of ticks have been recorded which covered approximately 120 species, including 10 species of Argasidae and over 100 species of Ixodidae [8]. Tick species were specific in different zones in China [9].

There were ten major tick-borne diseases reported in China, such as Tick-borne encephalitis (Forest encephalitis, TBE), Q-fever, Oriental spotted fever, North-Asia tick-borne spotted fever, Crimean-Congo hemorrhagic fever (Xinjiang hemorrhagic fever), Colorado fever, Tick-borne relapsing fever, Lyme borreliosis, tularemia and piroplasmosis. These diseases were mostly reported in northern and northeastern China in areas such as Xinjiang, Inner Mongolia, Heilongjiang, Jilin, Liaoning and Yunnan provinces [10].

The distribution of ticks and tick-borne pathogens varied in different provinces in China with uneven distribution in space and time. Lyme borreliosis is caused by *Borrelia burgdorferi sensu lato.* The first human case of Lyme borreliosis was reported in a forest region of Heilongjiang province in 1985 [11]. Up to date, human borreliosis cases have been confirmed in 29 provinces and 19 provinces have been indicated to be the natural foci. TBE, caused by the TBE virus (TBEV), was first reported in 1952 in China [12] and now mainly occurs in mountainous areas and forest regions of north China, such as Heilongjiang, Jilin, Xinjiang, Inner Mongolia. Q-fever, caused by the infection with *Coxiella burnetii*, is distributed in more than 20 provinces. The first case was discovered in 1950 in China and outbreaks occurred in Inner Mongolia, Sichuan, Xinjiang , Yunnan and Tibet [13]. Piroplasmosis caused by *Babesia* and *Theileria* infections were endemic in livestock in Qinghai, Gansu, Ningxia, Sichuan and Yunnan provinces [11]. However, human babesiosis is rarely reported in China. The first suspected case of human babesiosis was reported in 1982 in Yunnan province [14]. In 2012, a middle-aged woman in Zhejiang Province was reported infected with *Babesia microti*[15]. Although few human cases were also reported in Inner Mongolia and Taiwan [16,17], the epidemiological and transmission characteristics of babesiosis were unclear.

Human granulocytic anaplasmosis (HGA), an emerging infectious disease in China, is caused by *Anaplasma phagocytophilum*. The first human case of HGA was reported in Anhui province in 2006 [18], and then a series outbreaks occurred in Anhui, Tianjin, Shandong, Heilongjiang, Xinjiang and Hainan [19]. More recently, several outbreaks of TBDs, which may lead to organ failure and death have been reported in the central regions of China since 2007 [20]. Until 2011, more than 1000 cases had been reported in Henan province, and 98.75% cases were in Xinyang city and mainly occurred between April to October [21]. Most of patients were farmers and residents in the mountainous or hilly villages with history of tick bites [22]. In 2010, a new virus, isolated from blood samples of such patients from Henan province, was named as the severe fever with thrombocytopenia syndrome virus (SFTSV), which became another emerging TBD in China [23].

It has been reported that one tick species can transmit a variety of pathogens, and several kinds of TBDs often co-exist in the same natural foci [24]. Therefore, if humans or animals were bit by ticks with co-infections, it could result in a more complicated pathogenicity and worse prognosis. The potential threats of emerging pathogens as well as their co-infections due to the local social economic development and alteration of the natural environment will pose high risks to human health. For instance, the number of patients with fever of unknown origin is on the rise at peak activity period of ticks. This provides the hypothesis that there could be some unknown pathogens or co-infections in local ticks. Therefore, we investigated the fauna of ticks and potential pathogens and co-infections in Xinyang city which aimed to achieve a better understanding of distribution of tick species and tick-borne pathogens in central China.

## Methods

### Ethical clearance

Ethical and institutional approval documents were given by National Institute of Parasitic Diseases, Chinese Center for Disease Control and Prevention.

### Tick collection and identification

**Figure 1:**
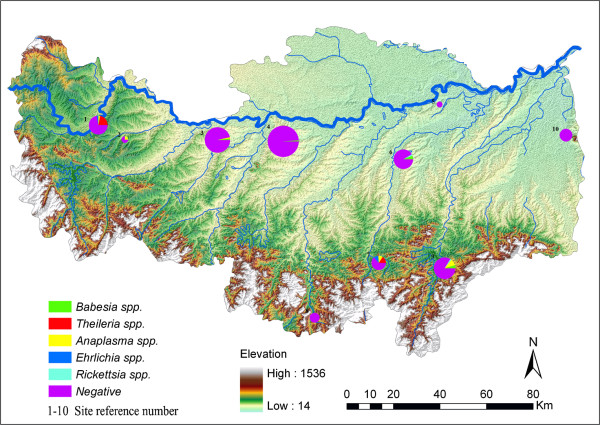
**Spatial distribution of various pathogens in sampling sites in Xinyang, Henan province, China.** (The size of the pie chart is proportional to the number of tested ticks in each sites, the numbers are shown in Table 2).

An investigation was conducted from April to December 2012 in 10 different villages located in 8 counties and 2 districts in Xinyang (Figure 1). The collection sites were determined by a method of random grid sampling, which was performed in ArcGIS. Ticks were collected once in each sampling site from the skin of domestic animals including sheep, cattle and dogs. The number of ticks collected from each individual animal was not more than 15. Ticks were counted and grouped according to their developmental stage. The species were identified based on morphologic criteria [25]. The specimens were kept frozen at -20°C with RNA*later* RNA Stabilization Reagent (Qiagen, Germany) and used for further molecular identification and detection of tick-borne pathogens.

### DNA and RNA extraction

Ticks were individually crushed with liquid nitrogen and plastic homogenizer using AllPrep DNA/RNA Mini Kit (Qiagen, Germany) for DNA and RNA extraction according to the handbook’s instructions. cDNA was synthesized from freshly extracted total RNA immediately by reverse transcription using OneStep RT-PCR Kit (Qiagen, Germany) also followed the handbook’s instructions. DNA, RNA and cDNA of ticks were stored at -80°C until use.

### PCR amplification and sequencing

In this study, each tick specimen was screened by PCR for both identification of tick species and detection of pathogens including *Babesia* spp.*, Theileria* spp.*, Leishmania infantum*, *Anaplasma* spp., *Ehrlichia* spp.*, Rickettsia* spp.*,* tick-borne encephalitis virus (TBEV) and *Borrelia burgdorferi sensu lato*. The tick species were confirmed by PCR using specific primers 16S + 1 (5′-CCGGTCTGAACTCAGATCAAGT-3′) and 16S-1 (5′-CTGCTCAATGA TTTTTTAAATTGCTGTGG-3′) [26]. One step PCR was used to detect *L. infantum*[27]. Nested PCR was used for detection of *Babesia* spp., *Theileria* spp.*, Anaplasma* spp., *Ehrlichia* spp.*, Rickettsia* spp.*, B. burgdorferi s. l.* and TBEV with protocols described in the references [28-32]. The target genes, specific primers, PCR methods used for testing different pathogens are listed in Table 1. Aliquot of double distilled water were included in all PCR runs to detect contamination. All PCR were carried out on a C1000 Touch™ Thermal Cycler (BIO-RAD, USA). PCR products were sent to Sangon Biotech (Shanghai, China) for sequencing in both directions. Sequences in this study were compared with sequences available in the NCBI database by BLAST analysis.

**Table 1:** Target genes, primers sequence, PCR methods used for pathogens identification

**Pathogens**	**Method**	**Target gene**	**Primers sequence (5′-3′)**	**Product size (bp)**	**Reference**
*Babesia/Theileria*	Nested PCR	18S rRNA	RIB-19(CGGGATCCAACCTGGTTGATCCTGC)	1700	[28]
			RIB-20(CCGAATTCCTTGTTACGACTTCTC)		
			BAB-F(ACCTCACCAGGTCCAGACAG)	430	
			BAB-R(GTACAAAGGGCAGGGACGTA)		
*Anaplasma/Ehrlichia*	Nested PCR	16S rRNA	Eh-out1 (TTGAGAGTTTGATCCTGGCTCAGAACG)	653	[29]
			Eh-out2 (CACCTCTACACTAGGAATTCCGCTATC)		
			Eh-gs1 (GTAATACTGTATAATCCCTG)	282	
			Eh-gs2 (TATAGGTACCGTCATTATCTTCCCTAC)		
TBEV	Nested PCR	Non-structural protein NS5	FSM1 (GAGGCTGAACAACTGCACGA)	357	[32]
			FSM2 (GAACACGTCCATTCCTGATCT)		
			FSM1i (ACGGAACGTGACAAGGCTAG)	251	
			FSM2i (GCTTGTTACCATCTTTGGAG)		
*Rickettsia* spp.	Nested PCR	groEL	Gro1 (AAGAAGGCGTGATAAC)	200	[30]
			Gro2 (ACTTCCGTAGCACC)		
			SF (GATAGAAGAAAAGCAATGATG)	250	
			SR (CAGCTATTTGAGATTTAATTTG)		
*B. burgdorferi s. l.*	Nested PCR	Flagellin	Outer1 (CTGCTGGCATGGGAGTTTCT)	730	[31]
			Outer2 (TCAATTGCATACTCAGTACT)		
			Inner1 (AAGGAATTGGCAGTTCAATC )	290	
			Inner2 (ACAGCAATAGCTTCATCTTG )		
*Leishmania infantum*	PCR	kDNA	RV1(CTTTTCTGGTCCCGCGGGTAGG)	183	[27]
			RV2(CCACCTGGCCTATTTTACACCA)		

### Statistical analysis

Differences in the numbers of collected ticks and positive rates of pathogens in different animal species and terrain types were tested by *χ*^2^-test, which was performed in SPSS 18.0.

## Results

### Tick identification

A total of 308 ticks were collected in 10 villages (range 3–89 ticks per site). Only two tick species were sampled. Both were Ixodidae. The most abundant species was *H. longicornis* (96.75%). The other one was *R. microplus* (3.25%)*.* 298 *H. longicornis* had been collected from all hosts species in all 10 villages, but only 10 *R. microplus* were collected from sheep and cattle in 3 villages (sampling site 1, 4 and 5). The majority of collected ticks were adult (female 86.69%, male 6.82%). Only a few of nymphs (5.84%) and larvae (0.65%) were sampled.

### Pathogen detection and identification

*Babesia* spp*., Theileria* spp.*, Ehrlichia* spp.*, Anaplasma* spp. and *Rickettsia* spp. were detected in 7 villages, and the positive rates were 1.95%, 3.25%, 0.97%, 2.92% and 0.65%, respectively. TBEV, *B. burgdorferi s. l.* and *L. infantum* were not detected in any ticks. There was no positive tick found in three villages (sampling sites 5, 8 and 10).

Piroplasms were the most frequently detected pathogen, the positive rate was 5.19%. The prevalence of detected pathogens in each sampling site was shown in Table 2 and Figure 1. In this study, four *Theileria* (*T. sergenti*, *T. orientalis*, *T. buffeli* and *T. luwenshuni*) and three *Babesia* (*B. gibsoni*, *B. canis vogeli* and *B. microti)* species were identified. *A. phagocytophilum*, *Rickettsia* sp. and *Ehrlichia* sp. were detected. The sequences of detected pathogens in this study were deposited in GenBank, and the GenBank accession numbers are shown in Table 3.

**Table 2:** Prevalence of detected pathogens in different sampling site

**Detected pathogens**	**Positive ticks and prevalence of pathogens in each sampling site**										
	**1**	**2**	**3**	**4**	**5**	**6**	**7**	**8**	**9**	**10**	**Total**
	**(n = 32)**	**(n = 4)**	**(n = 59)**	**(n = 89)**	**(n = 3)**	**(n = 35)**	**(n = 19)**	**(n = 9)**	**(n = 43)**	**(n = 15)**	**(n = 308)**
*Babesia* spp.	0	0	1 (1.69%)	1 (1.12%)	0	2 (5.71%)	0	0	2 (4.65%)	0	6 (1.95%)
*Theileria* spp.	7 (21.88%)	0	0	0	0	0	3 (15.79%)	0	0	0	10 (3.25%)
*Anaplasma* spp.	1 (3.13%)	1 (25.00%)	1 (1.69%)	0	0	0	2 (10.53%)	0	4 (9.30%)	0	9 (2.92%)
*Ehrlichia* spp.	1 (3.13%)	0	0	0	0	0	2 (10.53%)	0	0	0	3 (0.97%)
*Rickettsia* spp.	0	0	0	0	0	1 (2.86%)	0	0	1 (2.33%)	0	2 (0.65%)

**Table 3:** Detected pathogens in ticks collected from different hosts in different locations, and GenBank accession numbers in this study

**Pathogens (No. positive)**		**Ticks species**	**Animal species**	**Sampling site No.**	**GenBank accession No.**
*Theileria*	*T. buffeli* (2)	*H.longicornis*	Cattle	1	KJ715170, KJ715175
	*T. sergenti* (3)	*H.longicornis*	Cattle	1	KJ715171, KJ715173, KJ715174
	*T. orientalis* (1)	*H.longicornis*	Cattle	1	KJ715172
	*T. luwenshuni* (3)	*H.longicornis*	Sheep	7	KJ715167- KJ715169
*Babesia*	*B. canis vogeli* (3)	*H.longicornis*	Sheep, Dog	3,4,9	KJ715161, KJ715164, KJ715165
	*B. gibsoni* (2)	*H.longicornis*	Dog	6,9	KJ715162, KJ715166
	*B. microti* (1)	*H.longicornis*	Dog	6	KJ715163
*Rickettsia*	*Rickettsia* sp. (2)	*H.longicornis*	Sheep, Dog	6,9	KJ715194, KJ715195
*Ehrlichia*	*Ehrlichia* sp. (3)	*H.longicornis*	Sheep, Cattle	1,7	KJ715196- KJ715198
*Anaplasma*	*A. phagocytophilum* (9)	*H.longicornis*	Sheep, Dog, Cattle	1,2,3,7,9	KJ715199- KJ715207

Overall, 8.77% of ticks were tested positive for at least one pathogen. 8.72% of *H. longicornis* and 10% of *R. microplus* were detected positive. All the pathogens were detected in *H. longicornis,* and only one pathogen (*Theileria* spp.) was detected in *R. microplus*. The overall prevalence of pathogens in larvae, nymphs and adult ticks were 0.00%, 5.56% and 9.03%, respectively. There was no significant difference in the prevalence of these pathogens among different developmental stages of ticks (all *P*>0.05). However, there were significant differences in prevalence of these pathogens among host species and terrain types. Prevalence of these pathogens in ticks collected from sheep, dogs and cattle were 9.23%, 4.17% and 26.47%, respectively. The positive rate of pathogens in ticks collected from cattle was 2.87 times (*χ*^2^ = 7.17, df = 1, *P* < 0.05) and 6.35 times (*χ*^2^ = 17.73, df = 1, *P* < 0.05) more than that from sheep and dogs, respectively. Prevalences of these pathogens in ticks in mountainous, hilly and plain areas were 20.56%, 2.03% and 3.77%, respectively. The positive rate of pathogens in ticks collected from mountainous area was 5.45 times (*χ*^2^ = 7.83, df = 1, P < 0.05) and 10.14 times (*χ*^2^ = 24.12, df = 1, P < 0.05) more than that from plain and hilly areas respectively. The results are displayed in Table 4.

**Table 4:** Comparison of the differences of collected ticks and positive rates of pathogens among ticks life stage, host species and terrain types

**Groups**		**Sampled ticks**	**Positive ticks**		***χ***^**2**^	**DF**	**RR (95% CI)**	**P**
		**N**	**n**	**%**				
Life stage	Larvae	2	0	0.00	0.12	1	-	0.7324
	Nymphs	18	1	5.56			-	
	Larvae	2	0	0.00	0.20	1	-	0.6561
	Adult	288	26	9.03			-	
	Nymphs	18	1	5.56	0.25	1	1.00	0.6144
	Adult	288	26	9.03			1.62 (0.23-11.30)	
Terrain feature	Hilly	148	3	2.03	24.12	1	1.00	0.0000
	Mountainous	107	22	20.56			10.14 (3.12-33.02)	
	Hilly	148	3	2.03	0.49	1	1.00	0.4836
	Plain	53	2	3.77			1.86 (0.32-10.84)	
	Plain	53	2	3.77	7.83	1	1.00	0.0051
	Mountainous	107	22	20.56			5.45 (1.33-22.31)	
Hosts	Dogs	144	6	4.17	2.85	1	1.00	0.0911
	Sheep	130	12	9.23			2.22 (0.86-5.73)	
	Dogs	144	6	4.17	17.73	1	1.00	0.0000
	Cattle	34	9	26.47			6.35 (2.42-16.64)	
	Sheep	130	12	9.23	7.17	1	1.00	0.0074
	Cattle	34	9	26.47			2.87 (1.32-6.24)	

DF = degrees of freedom; RR = risk ratio; CI = confidence interval.

### Co-infections

Out of 27 positive ticks, 3 ticks (11.11%) were found co-infected with two pathogens. One co-infection detected was *B. microti* (KJ715163) with *Rickettsia* sp. (KJ715194) in one *H. longicornis* tick collected from a dog in sampling site 6. The other two co-infections were *T. luwenshuni* (KJ715167) with *Ehrlichia* sp. (KJ715196) and *T. luwenshuni* (KJ715168) with *A. phagocytophilum* (KJ715199) in two *H. longicornis* ticks which were both collected from sheep in sampling site 7.

### Spatial distribution

The spatial distribution of pathogens is shown in Figure 1. The prevalence and diversity of pathogens were much higher in the middle elevation regions, which mostly were mountainous areas (sampling site 1,2,7 and 9). Relatively, there were lower prevalence rates and fewer species of pathogens detected in low elevation regions, which mostly were plain or hilly areas (sampling site 3,4,5,6 and 10) as well as in the high elevation region, which is mountain top area (sampling site 8). The geographical locations of co-infections were adjacent to each other (sampling site 6 and 7).

## Discussion

Xinyang city is located at the sub-tropical region of China. The western, southern and central regions are mountainous or hilly areas, and the north regions are plain areas (Figure 1). Relatively high humidity and temperature during the summer provide a suitable environment for the development and reproduction of ticks.

In this study, we found that *H. longicornis* was the dominant tick species in Xinyang which didn’t have any host specificity. These results are consistent with previous studies [33-36], which suggested that *H. longicornis* could play an important role as the reservoir host for various pathogens and the source of disease in this area. Only a few *R. microplus* were collected in this study, but its distribution was similar to previous studies [37,38]. Moreover, previous study documented the existence of *O. lahorensis* and *I. persulcatus* in this area [39], however no samples of *O. lahorensis* and *I. persulcatus* were collected in this study. This could be attributed to only one transmission season as well as the limited number of host species that were taken into account.

The results of this study have demonstrated two interesting facts about infections in ticks which were correlated to the impacts of local environment and social activities. First, the positive rates of pathogens in ticks were significantly higher in the mountainous areas than those in the plain areas. This is probably because of the diversity and larger population size of host animals in mountainous areas. Second, the positive rate of ticks was significantly higher in ticks collected from cattle and sheep. This situation is potentially related to local animal husbandry. In fact, sheep husbandry was more common in the rural area, but most farmers rarely neutralized parasites and sheepfolds were kept close to the villagers’ house for the purpose of anti-theft. It was reported in the Henan Statistical Yearbook 2012 that a total of 818.4 thousands of sheep were raised in Xinyang. Therefore, all these factors could pose high risks of exposure to humans resulting in human infections with those pathogens.

Overall*, Anaplasma* spp. and *Babesia* spp. were distributed in continuous areas with overlapped regions. The other three pathogens were distributed in separate foci respectively. This is the first report of *Rickettsia* spp. in ticks collected from domestic animals in this region. *R. typhii* and spotted fever group rickettsiae had been detected in rodents caught in this area [40], although no human cases have been reported yet. In fact, human ehrlichiosis (HE) had been clinical diagnosed in local farmers that had evidence of tick bites in Xinyang [41]. This finding suggested that Xinyang would potentially be an endemic area of human ehrlichiosis.

The positive rate of *Theileria* spp. was especially high in ticks collected from cattle and sheep. Given that theileriosis was endemic in animals in Henan province [42,43], as well as in other regions [44,45], it could pose a high risk of exposure and infection to livestock and increase the economic burden on the breeding industry and farmers in this region. Although *Babesia* spp. infection rate was not the highest in this region, it is still higher than in other areas [46]. The positive rate was higher in ticks sampled from dogs, and similar result was reported in Thailand [47]. In this study, we found one tick infected with *B. canis vogeli* which was collected from a sheep, and one tick infected with *B. microti* which was collected from a dog. Generally speaking, *B. canis vogeli* infections were often detected in dogs, and so far there were no reports about its infection in sheep. These findings warrant further studies. However, *B. microti* had been detected in many kinds of wild and domestic animals including dogs, and could be the cause of human babesiosis as well. Babesiosis had been detected in livestock in China including Henan province [48-53], and human babesiosis had been diagnosed in some province in mainland China recent years [54,55]. Although there has been no report of human babesiosis in Xinyang yet, we suspected that some cases have been misdiagnosed as *Plasmodium* infection [56], since *Plasmodium* spp. was once heavily endemic in this area [57,58]. It would be even more difficult to differentiate *Babesia* spp. from *Plasmodium* spp. when a co-infection had occurred.

A new finding was that *Babesia* spp. co-infected with *Rickettsia* spp. in ticks in Xinyang region. The results also indicated that *Theileria* spp. might be more likely to co-exist with other pathogens in ticks. The spatial distribution of those pathogens suggested that humans and animals in the region were at the higher risk of exposures to co-infections.

In recent years, great progress had been made on identification of tick-borne disease (TBD) vectors, hosts and evaluating the impacts of TDBs to humans. However, it’s still necessary to carry out more studies on co-infections. So far as we know, there were only several reports documented that *B. burgdorferi s. l.* co-infected with other pathogens in ticks in north-eastern China where Lyme disease is known to be endemic [59-62]. Ticks co-infected with multiple pathogens greatly increased the risk of co-infections to humans, which would result in more complex clinical manifestation and could be misdiagnosed. Although there was no reports of co-infections of tick-borne pathogens in humans in China as of yet, great concern had been raised because the pathogens might share common tick vectors and reservoir hosts, which means transmission of co-infections to humans could be quite possible.

## Conclusions

Both human and animal pathogens occur in ticks in the study areas. Most of the tick species lack host specificity [63]. The impacts of global climate change, increased population mobility, decreased natural host populations, host-switching behavior of ticks [64] could lead to the outbreaks and endemic of tick-borne zoonoses once these public health threats transfer to humans. Further studies are needed to estimate the impacts to local residence and animal husbandry by these vectors and pathogens and to establish effective measures to control the vector ticks.

## Competing interests

The authors declare that they have no competing interests.

## Authors’ contributions

ZC conducted field sampling, performed tick species identification and the laboratory work, generated experimental data, and wrote the manuscript. QL and X-NZ had a substantial role in conception of the study, guidance of the practical work and writing the manuscript. J-QL and B-LX helped with sample collection. SL and SX helped with statistical analysis and contributed to the manuscript. All authors read and approved the manuscript.
